# Impact of the Tiloop Bra mesh in CT images and dose delivery in breast radiotherapy

**DOI:** 10.1120/jacmp.v13i2.3667

**Published:** 2012-03-08

**Authors:** Cristina Camacho, Jose Perez‐Calatayud, Françoise Lliso, Vicente Carmona, Alejandro Tormo, Facundo Ballester, Ma Carmen Pujades

**Affiliations:** ^1^ Radiotherapy Department La Fe University Hospital Valencia Spain; ^2^ Radiotherapy Department Hospital Clínica Benidorm Benidorm Alicante Spain; ^3^ Atomic, Molecular and Nuclear Department University of Valencia Burjassot Spain

**Keywords:** protheses, mesh, titanization, metallic artifact

## Abstract

A new titanized breast mesh, TiLOOP Bra, is currently available for implantation in patients who require radiotherapy. The purpose of this work is to study the dosimetric effect of the presence of a TiLOOP Bra mesh on breast radiation treatment and radiographic imaging. The dosimetric effects have been measured for three X‐ray energies: 1.25 MeV, 6 MV and 18 MV, using radiochromic films placed at three different depths. These depths are representative of mesh location in breast during the radiotherapy treatment and hence, are of interest in this study. In order to assess the disturbance in a radiographic image, different computed tomographic (CT) studies of the mesh have been performed. The absorbed dose differences with and without the mesh are less than 1%. No metallic artifacts have been observed in radiographic images associated with the mesh, nor significant disturbances in Hounsfield Units. The TiLOOP Bra mesh does not disturb the dosimetry of a typical radiotherapy treatment and its influence in the quality of the CT scan required for planning is negligible.

PACS number: 87.55.‐x

## I. INTRODUCTION

Effects on the radiotherapy treatment of prostheses partially designed with high‐density materials, like actual hip prostheses, are well documented.^(^ ^1^ ^,^ ^2^ ^,^ ^3^ ^)^ In relation to this issue, a new type of implant has become available on the market. It consists of a mesh which takes advantage of titanization technology, ensuring an appropriate biocompatibility. This new material has already been used in hernia surgery^(4)^ (TiMESH and TiLENE by PFM Medical, Nürnberg, Germany), skull,^(5)^ and breast.

Concerning the breast cases, the mesh TiLOOP Bra (PFM Medical) is designed to be used after reconstructive surgery, to reinforce the mammary implant. It consists of a submillimeter thick mesh of polypropylene totally coated with a very light (30–50 nm thick) layer of titanium. It is fixed to the caudal and lateral end of the pectoral muscle, being its lower part stitched to the inframammary fold during the surgical procedure. Therefore, the mesh is located within the first centimeter deep relative to the skin.

There are prospective randomized trials which demonstrate the important role of adjuvant radiotherapy after mastectomy in the management of high‐risk breast cancer.^(^ ^6^ ^,^ ^7^ ^,^ ^8^ ^)^ In those patients, after mastectomy and subsequent TiLOOP Bra mesh implantation, a postoperative radiotherapy (PORT) is required, in which the target volume and its surroundings (including the titanized mesh) will be irradiated during the radiotherapy treatment. Due to the presence of a certain thickness of metal, differences might be found between prescribed and delivered dose. For example, tissue expanders are traditional prostheses after breast surgery, usually designed with metallic ports. It has been shown that this metal part causes significant changes in the dose prescribed, since photon beam attenuation is of the order of 20%–30% for 6 MV clinical beams and 16% for 15 MV beams.^(^ ^9^ ^,^ ^10^ ^)^ In addition, when a filtered back‐projection method is used, high‐density objects cause streak artifacts on computed tomography (CT) images. These artifacts can disturb the target volume and other tissues' delineation. Even more, inaccuracies in the conversion of the Hounsfield Units (HU) from a CT image into relative electron density (required by the treatment planning system (TPS) to calculate dose distributions) can also compromise the results in these calculations. This is because the commercial TPS algorithms do not predict correctly the dose near high‐density objects. Finally, inaccuracies might also arise from image distortion.

The main purpose of this study is to investigate the dosimetric impact of the titanized mesh TiLOOP Bra when it is irradiated during breast radiotherapy treatments. For this, in‐phantom measurements have been done to determine how dose values are affected by the mesh. In addition, the influence of the mesh on both the quality and the perturbation of the HU of a radiographic image that is needed for the treatment planning has been evaluated.

## II. MATERIALS AND METHODS

**Figure 1:**
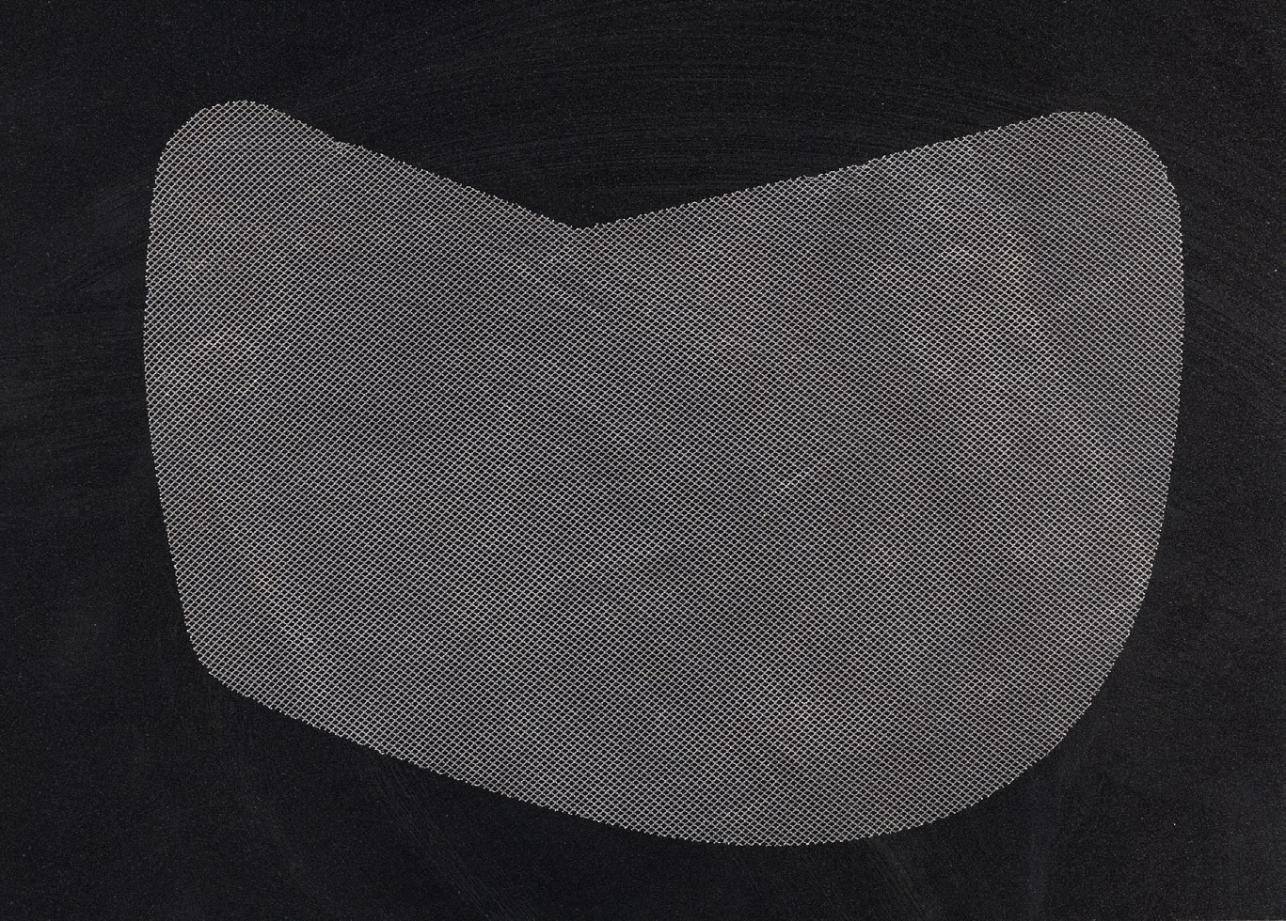
Geometric characteristics of the TiLOOP Bra mesh.

The TiLOOP Bra mesh for breast reconstructions studied in this work is composed by a covalent bonding of polypropylene ${\left(-(C_{3}H_{6}\right)}_{n}-)$ and a titanium dioxide surface. In the titanization process, the titanium is introduced in gaseous form; therefore, it reaches all parts of the polypropylene implant. As a consequence, the entire surface, including gaps between complex shapes, is completely coated with an ultralight layer of titanium. This layer ranges between 30 nm and 50 nm. TiLOOP Bra mesh is commercialized in different versions, depending on, basically, their characteristics and sizes. For each individual size (small, medium, and large), three different types are available. This information is summarized in Table 1, Table 2, and Fig. 1. The differences in size are due to the breast weight the mesh has to support: less than 200 g, between 200 and 350 g, and more than 350 until 500 g, for small, medium, and large size, respectively. Meanwhile, the titanized surface thickness is the same in the three versions. The particular mesh used in this study is medium size, and its total thickness is approximately 0.20 mm. The titanization layer represents, therefore, the 0.015%–0.025% of the total mesh thickness.

**Table 1:** Characteristics of different types of the TiLOOP Bra mesh.

	*Extralight*	*Type of Mesh Light*	*Strong*
Weight (g/m^2^)	16	35	65
Strength (DIN EN ISO 5084) (mm)	0.20	0.30	0.45
Pore size (mm)	≥1	≥1	≥1
Filament diameter, dtex (μm)	30 (65)	58 (90)	103 (120)
Porosity 2D	73	61	53
Porosity 3D	91	87	82
Physiological elasticity at 16N	23	20	8
Tensile strength (grab test) (N)	37	61	142

**Table 2:** Geometrical dimensions of the TiLOOP Bra mesh.

*a*	*Size (mm) b*	*c*
Small 195	95	20
Medium 215	115	140
Large 235	135	160

The effects of the mesh on absorbed dose were evaluated for three different clinical beams: a Co−60 unit whose mean energy is 1.25 MeV, and 6 and 18 MV from a linear accelerator (Precise SLi20, Elekta, Stockholm, Sweden). The dose distribution has been measured for the three energies as follows: the mesh was placed at 5 mm depth, fixed perpendicularly to the central beam axis. Dose profiles were measured in three regions at different depths placing three radiochromic film EBT2 sheets (GAFCHROMIC, International Specialty Products, Wayne, NJ) identified with batch A09171002, into a water equivalent phantom material (RW3, PTW‐Freiburg, Freiburg, Germany). Two radiochromic films were situated above and below the mesh just in contact with the mesh. In addition, a third film was situated at 5 cm depth from the surface of the phantom. The source‐to‐phantom surface distance was different depending on the treatment unit: 80 cm in Co−60 and 100 cm in the Precise SLi20. Meanwhile, the field size was constant for all the measurements: 20cm×20cm at the isocenter plane.

Radiochromic films have been calibrated in the Co−60 unit (Theratron Phoenix, Theratronics International Limited, Kanata, Ontario, Canada) in the range from 0 to 800 cGy fitted to a 4th degree polynomial. Films are digitized prior to irradiation and 24 hours after, to subtract, pixel by pixel, the pre‐exposure pixel values from the postexposure ones. This background subtraction, performed using an in‐house software program, reduces inaccuracies in scan measurements. For the digitization procedure, an EPSON Expression 10000XL Photo flatbed scanner (Seiko Epson Corp., Nagano‐Ken, Japan) is used. All film pieces are scanned in transmission mode four consecutive times at a resolution of 100 dpi and 48 bit colour. The final stored image is calculated as the average of those four images. The red channel of the final image is extracted and processed. DoseLab software (Mobius Medical System, LP, Houston, TX) was used for image analysis for raw signal, as well as dosimetry.

A dose of 1.8 Gy was prescribed at 5 cm depth, just where the third film was placed. This is a representative dose of breast treatment at our institution. With the same setup described above, five measurements at each energy were performed. For each energy, data were taken in two situations, with and without the mesh, to obtain the possible differences in the absorbed dose between both cases. It must be said that the setup used was chosen to mimic closely representations of breast geometry. The position of the two films just in contact with the mesh (at 5 mm depth) could represent the typical mesh depth in the breast. Therefore, one film is used to estimate the effects of backscattered electrons from the mesh, and the other one measures the possible photon beam attenuation. Meanwhile, the third film was placed at the mid‐depth of a typical breast volume to study the effects of photon beam attenuation.

**Figure 2:**
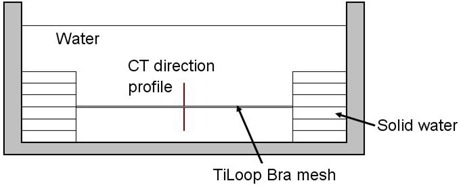
Experimental setup to evaluate the influence of TiLOOP Bra mesh in Hounsfield Units in a CT scan.

To assess the disturbance introduced by the titanized mesh in a radiographic image, some experiments were done. First, an in‐house phantom was manufactured using an inside breast implant made of cohesive gel. This implant was completely covered with the mesh and well fixed on a homogeneous polystyrene phantom, which could represent the thorax of a patient. In addition, the top of the mesh set was covered with a bolus (5 mm thick) in order to simulate water equivalent tissue all around the implant surface. All this was placed in an axial computed tomography (CT‐SYTEC, General Electric, Milwaukee, WI) and scanned with the following parameters: 120 kVp, 100 mA, and 5 mm slice thickness. The CT image was examined to discover the presence of visual metallic artifacts around the surface covered by the mesh. In the second procedure, the mesh was immersed into a tank totally filled with water (Fig. 2). It was laterally fixed to be parallel to the water surface. A new CT study was performed, with the same setup as before. Profiles of HU values, along the perpendicular direction of the mesh, have been analyzed with ImageJ software (v1.44, National Institutes of Health, Bethesda, MD) in a region of 1 cm above and below the mesh position.

## III. RESULTS & DISCUSSION

The dosimetric impact of the TiLOOP Bra titanized surface, understood as the difference in absorbed doses due to the presence of the mesh at various depths and energies, has been measured and results are presented in Table 3. The mean values of the absorbed dose of five measurements and their standard deviations have been obtained for each energy. Using these mean values, the dose relative differences between those cases where the mesh is present and those where the mesh has been removed have been evaluated.

**Table 3:** RF measurement results. Dose values correspond to the mean of five measurements, and the uncertainty is the standard deviation of the mean (k=1). Difference uncertainties are obtained by quadratic propagation of uncertainties (k=1).

		*With mesh*, $D_{m}$	*Dose (cGy) Without mesh, D*	*Difference*, $\frac{D_{m}-D}{D}\%$
6 MV	Above	184.8±2.5	183.6±2.7	0.6±2.0
	Below	183.4±2.7	183.6±2.7	−0.1±2.1
	5 mm depth	185.4±2.6	185.8±3.9	−0.2±2.5
18 MV	Above	138.4±2.6	138.1±2.9	0.2±2.8
	Below	137.8±3.0	138.1±2.9	−0.2±3.0
	5 mm depth	183.7±1.1	183.8±1.9	−0.1±1.2
Co−60	Above	239.7±1.6	238.0±2.2	0.7±1.1
	Below	234.5±1.7	238.0±2.2	−1.5±1.2
	5 mm depth	179.9±0.7	180.6±1.9	−0.4±0.6

According to the literature, assigning a specific absolute uncertainty to radiochromic film measurements is a controversial issue. Nevertheless, it could be estimated that absolute absorbed doses measured using EBT2 films can be given with an overall uncertainty not less than 4%–6%.^(^ ^11^ ^,^ ^12^ ^)^ In our case, with five measurements, the standard deviation of the mean (type A uncertainty) varies in the range 0.5%–2%, which in turn gives a relative uncertainty of the differences of about 2%. Taking into account the relative differences and their uncertainties presented in Table 3, the conclusion of this study is that the presence of the mesh does not perturb appreciably the dose distribution.

As has been noted,^(^ ^9^ ^,^ ^10^ ^)^ dosimetric effects of some traditional breast prostheses, such a metallic ports in tissue expanders, reach much higher levels when clinical beams with similar energies to those studied here are used. As a result, the improvement by using TiLOOP Bra mesh in the breast surgery reconstruction has been clearly demonstrated.

Other significant aspects relative to the implication of pore sizes on dosimetry, such as photoneutron activation of Ti (a phenomenon observed at high photon energies such as 18 MV) or the impact for clinical electron beams (also used in breast radiotherapy), have not been included in this study. The initial scope was only to show the general dosimetry improvements of TiLOOP Bra mesh compared with traditional kinds of prostheses in which both aspects — photoneutron activation and beam attenuation — would be more pronounced. A second phase study is being developed using Monte Carlo (MC) simulations for the modeling of external radiotherapy photon and electron beams, in order to evaluate microdosimetry aspects and the influence on electron beams.

**Figure 3:**
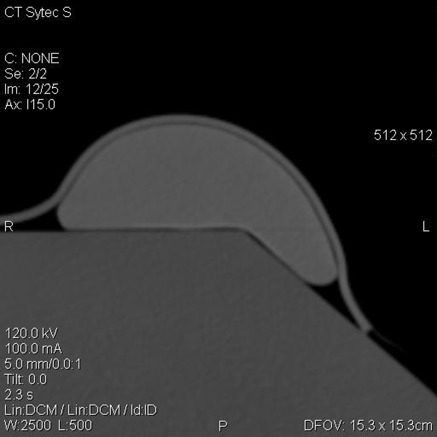
Slice of the CT study performed using the breast implant phantom covered with the TiLOOP Bra mesh and 5 mm bolus. No metallic artifacts are observed.

Regarding the influence of the mesh on the quality of a radiographic image, the result of the first experimental setup based on an anatomical phantom is that TiLOOP Bra mesh does not cause streak artifacts in CT images. Figure 3 shows one of the slices acquired in the CT scan with 2500 Windowing and 500 Grey Level values. The bolus can be well appreciated covering both the gel implant and the polystyrene phantom. However, due to its minimal thickness, the mesh placed just on the gel implant cannot be appreciated in the image. As can be seen in Fig. 3, no metallic artifacts are observed in the image, neither in the slice shown nor in the others.

**Figure 4:**
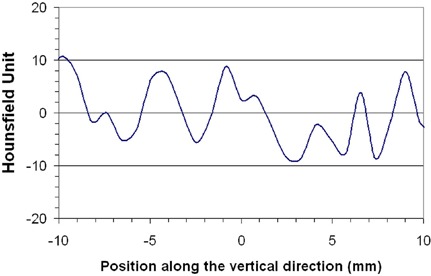
Representation of a CT image profile indicated in Fig. 2.

In the second experimental setup, some profiles have been taken of different slices in a perpendicular direction to the mesh to evaluate possible disturbance in the HU due to the high density component of the mesh. Figure 4 shows the result obtained for one profile. The order axis shows a reduced HU scale, from HU=20 to HU=−20, to include the HU values extracted from the profile. Meanwhile, the origin in the abscissa axis corresponds to the position of the mesh. All the obtained HU values are in the region of −10HU to +10HU, as it is expected for water (HU=0±5). In terms of electron density, the maximum difference found, relative to the water value, has resulted to be less than 0.7%. In addition, there are no high HU values, as is expected for high‐density materials like titanium.

## IV. CONCLUSIONS

In this study, the dosimetric impact of the titanized mesh TiLOOP Bra when it is irradiated during breast radiotherapy treatments has been investigated. TiLOOP Bra mesh impact on the absorbed dose has been evaluated for three clinical photon beams by in‐phantom radiochromic film measurements. The differences in dose values with and without the mesh is compatible with zero, providing uncertainties (2%) are taken into account. The dosimetric disturbance of the TiLOOP Bra mesh in breast radiotherapy treatment is much smaller than the disturbance observed with other traditional breast prostheses.

In addition, the influence of the mesh in both the quality and the perturbation of the HU of a radiographic image that is needed for the treatment planning has been also evaluated. It has been determined that the influence of the mesh in the quality of a radiographic image is negligible. The quality of the CT image is not affected (since metallic artifacts cannot be detected) and changes in HU values have not been observed in the presence of the mesh.

In conclusion, TiLOOP Bra mesh does not disturb the dosimetry of a typical breast radiotherapy treatment, and its influence in the quality of the CT scan required for planning is negligible.

## ACKNOWLEDGMENTS

This study has been supported by Sumedex S.A. This study was supported in part by Generalitat Valenciana, (Project PROMETEO2008/114) and Ministerio de Ciencia e Innovación, Spain (Project No. FIS2010‐7007).
